# Continuation of tropical Pacific Ocean temperature trend may weaken extreme El Niño and its linkage to the Southern Annular Mode

**DOI:** 10.1038/s41598-019-53371-3

**Published:** 2019-11-19

**Authors:** Eun-Pa Lim, Harry H. Hendon, Pandora Hope, Christine Chung, Francois Delage, Michael J. McPhaden

**Affiliations:** 1 Bureau of Meteorology, Melbourne, VIC 3000 Australia; 2 Pacific Marine Environmental Laboratory, National Oceanic and Atmospheric Administration, Seattle, WA USA

**Keywords:** Climate sciences, Projection and prediction

## Abstract

Observational records show that occurrences of the negative polarity of the Southern Annular Mode (low SAM) is significantly linked to El Niño during austral spring and summer, potentially providing long-lead predictability of the SAM and its associated surface climate conditions. In this study, we explore how this linkage may change under a scenario of a continuation of the ocean temperature trends that have been observed over the past 60 years, which are plausibly forced by increasing greenhouse gas concentrations. We generated coupled model seasonal forecasts for three recent extreme El Niño events by initialising the forecasts with observed ocean anomalies of 1 September 1982, 1997 and 2015 added into (1) the current ocean mean state and into (2) the ocean mean state updated to include double the recent ocean temperature trends. We show that the strength of extreme El Niño is reduced with the warmer ocean mean state as a result of reduced thermocline feedback and weakened rainfall-wind-sea surface temperature coupling over the tropical eastern Pacific. The El Niño-low SAM relationship also weakens, implying the possibility of reduced long-lead predictability of the SAM and associated surface climate impacts in the future.

## Introduction

The Southern Annular Mode (SAM) is the leading mode of variability of the Southern Hemisphere (SH) extratropical circulation on weekly and longer timescales that describes a meridional vacillation of the eddy-driven jet and associated storm track^1–5^. The spatial pattern of the positive polarity of SAM (high SAM) is characterised by a nearly zonally symmetric annular pattern of positive anomalies of pressure/geopotential height in the SH midlatitudes and negative anomalies in the Antarctic region, associated with a poleward shift of the eddy-driven jet and storm track^3^. The negative polarity of SAM (low SAM) is characterised by an equatorward shift of the eddy-driven jet and associated storm track. SAM, which is intrinsic to the troposphere, exhibits a decorrelation time of about two weeks^6^ and can be realistically simulated in an atmospheric general circulation model forced with climatological sea surface temperatures (SSTs) at the lower boundary^7,8^. In addition to the well established trend toward the higher polarity phase of SAM in response to the Antarctic ozone depletion, year-to-year variations of seasonal mean SAM during spring and summer can be promoted by the El Niño-Southern Oscillation (ENSO)^4,9–15^ and by downward coupling of anomalous conditions in the Antarctic stratospheric polar vortex that develops as early as austral winter^16–18^.

The relationship between ENSO and SAM is particularly important for a long-lead prediction of  SAM and its surface climate anomalies because ENSO can be skillfully predictable at lead times of 2–3 seasons and beyond^4,19^. During austral spring and summer, between 10–36% of the variance of SAM is explained by its relationship with eastern Pacific-type (EP) ENSO^4,9,10,13^. The warm phase of ENSO (El Niño) is associated with low SAM and the cold phase of ENSO (La Niña) is associated with high SAM. Seasonal mean SAM during austral spring and early summer is predictable with an atmosphere-ocean coupled forecast system at up to 6 month lead time, and the long-lead predictive skill is likely to be attributed to the relationship of SAM with eastern Pacific ENSO^4^.

The predictability of SAM stemming from ENSO leads to predictability of SH extratropical atmosphere, ocean and sea-ice variations in regions which are strongly influenced by SAM^13,20–25^. This is in addition to the predictability arising from direct impacts of ENSO^26^. Because of the potential benefits of predicting extratropical climate as a result of the co-variation of SAM with ENSO, it seems natural to question how the ENSO-SAM relationship will change in a future climate given that there has been much focus on how ENSO and its impacts might change in warmer climte^27,28^. Unfortunately, there is great uncertainty in how this relationship may change in the future because, for instance, the models used in the Climate Coupled Model Intercomparison Project (CMIP5)^29^ fall significantly short in simulating the observed linkage between ENSO and SAM in the current climate, demonstrating large inter-model spread all year round^30^. Therefore, an idealised model experiment would be useful to explore how SAM might respond to ENSO in a future climate.

In this study, we specifically focus on the response of SAM to extreme El Niño in a future waremer climate because characteristics of extreme El Niño and extreme La Niña^31^ are not entirely symmetric and their impacts on the atmosphere are not exactly opposite to each other^32,33^. This focus on extreme El Niño is also to increase the signal in the model experiments. We explore our research question by superimposing 1982, 1997 and 2015 El Niño oceanic conditions on a hypothetically warmer mean state, which we derive as a continuation of the observed ocean temperature trends since 1960. This is done by using a series of seasonal forecast sensitivity experiments with the global coupled model seasonal prediction system POAMA (Predictive Ocean and Atmosphere Model for Australia)^34^.

**Figure 1:**
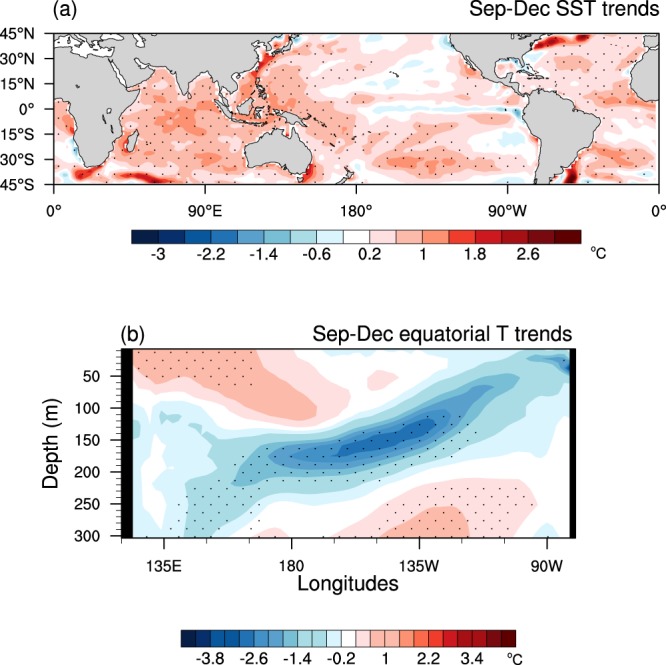
Observed sea surface and Pacific ocean subsurface temperature trends. Trends in September to December mean of (**a**) sea surface temperatures (SSTs) and (**b**) equatorial Pacific ocean subsurface temperatures estimated over 1960–2014 using PEODAS ocean reanalysis data (see Methods). The colour shading interval is 0.4 °C, beginning at +/−0.2 °C, for both (**a**) and (**b**). The stippling indicates statistical significance of the trends at the 5% level (i.e. *p* < 0. 05), which was assessed by a two-sided Student t-test with 55 samples. Calculation of the SST and subsurface temperature trends over 54 years 55 times, leaving a year out each time (i.e. cross-validation) confirms the robustness of the trend patterns (not shown).

The pattern of the observed ocean surface temperature trends of 1960–2014 is characterised by significant surface warming trends in the tropical western Pacific and Indian Oceans together with a slight surface cooling trend along the equatorial eastern Pacific^35–39^ (Fig. 1a). In the subsurface along the equator, there has been a significant cooling trend along and below the thermocline in the equatorial Pacific (Fig. 1b). This overall trend pattern is often described as a “La Niña-like” mean state change for convenience^40^. This trend pattern and magnitude in SSTs is well captured by the 1^st^ empirical orthogonal function (EOF) eigenvector of decadally smoothed SST variability, which is well separated from the 2^nd^ EOF eigenvector that represents the Inter-decadal Pacific Oscillation (IPO)/Pacific Decadal Oscillation (PDO)^41^ (Supplementary Fig. S1). This observed trend pattern is distinctively different from the trend patterns simulated by the CMIP multi-model mean historical and future projections in responses to increasing greenhouse gases^42^. The projected trend shows greater warming over the tropical eastern Pacific compared to its surrounding oceans and flattening of the thermocline in the equatorial Pacific^32,42,43^ (i.e. a “El Niño-like” mean state change).

An El Niño-like mean state change in response to increasing greenhouse gases is explained by weakening of the Pacific Walker circulation and the zonal SST gradient in the tropical Pacific, consistent with the energetic and hydrological balances under increasing greenhouse gas forcing^44–46^. The amplitude of El Niño is closely linked to the degree of mean warming in the eastern equatorial Pacific in the CMIP5 models, the majority of which show El Niño-like mean state change patterns^42^. Consequently, a significant increase in the frequency of extreme El Niño is projected in the 21^st^ century compared to the 20^th^ century^28^. However, the fidelity of this El Niño-like simulated mean state change in response to greenhouse gas forcing and its potential impact on ENSO characteristics have been vigorously debated^36,39,47–51^. The El Niño-like mean state change in some models has been attributed to systematic model biases such as a too regular and symmetric El Niño-La Niña cycle together with ocean mixed layers that are too deep in the tropical Pacific Ocean^51,52^; too weak inter-basin teleconnections in the tropical oceans^53^; and a too-cold cold tongue in the equatorial Pacific with too high relative humidity and too low wind speed^39^. On the other hand, the ocean dynamical thermostat mechanism^40,54^, the non-linear ENSO warming suppression mechanism^55^, and/or the inter-basin warming contrast mechanisms^56,57^ suggest that equatorial eastern Pacific SST would warm more slowly than the equatorial western Pacific SST as the Earth warms. Therefore, the possibility cannot be ruled out that the observed La Niña-like ocean temperature trend has been in part forced by global warming^39,56,58^, and it may continue into the future. Thus, we suggest that it is a valid and valuable question to address how the characteristics and teleconnection of extreme El Niño will change in the future if the observed La Niña-like long-term mean state warming continues, a scenario which has not yet been given much attention.

We address this premise by conducting four forecast sensitivity experiments whereby the ocean initial conditions are altered in a manner following the approaches of earlier studies using POAMA^59,60^. These experiments are summarised in Table 1. The present climate El Niño experiment (**pElNiño**) was conducted by initialising the coupled forecast model POAMA with observed ocean initial conditions for 00 UTC 1 September of 1982, 1997 and 2015 that represent the developing stages of the three extreme El Niños observed in the modern instrumental record^43^. We also initialised POAMA with the mean ocean conditions for 00 UTC 1 September computed over 1981–2013 in order to produce a set of climatological forecasts for the present climate (the present climatology experiment; **pClim**). To explore changes to extreme El Niño events and their linkages to SAM in the climate warmed up by enhanced observed ocean temperature trends, we computed the trends in ocean temperatures and salinity at all available vertical levels, latitudes, and longitudes on 00 UTC 1 September for the period of 1960–2014. We then doubled their magnitudes in order to produce a stronger impact in our modeling framework (Supplementary Fig. S2). We added these doubled trends to the observed ocean initial conditions used in pElNiño to generate forecasts of El Niño that occur with a warmer ocean mean state (the warmer climate El Niño experiment; **wElNiño**). Finally, we generated the climatological forecasts of the warmer climate by adding the doubled ocean temperature and salinity trends to the climatological ocean initial conditions used in pClim. We refer to this as the warmer climatology experiment, **wClim**. We denote present and warmer climate predicted El Niño with respect to their respective present and warmer climatologies as **pElNiño’** and **wElNiño**’, respectively.

**Table 1:** Design of POAMA forecast sensitivity experiments.

	Ocean Initial Conditions	Atmosphere & Land Initial Conditions	CO_2_	Ozone
pElNiño	1982, 1997, 2015	Thirty-three different conditions drawn from 1981–2013	345 ppm	Monthly climatology
wElNiño	pElNiño+2xTR			
pClim	climatology of 1981–2013			
wClim	pClim + 2xTR			
pElNiño’	pElNiño − pClim			
wElNiño'	wElNiño − wClim			

All the atmosphere, land and ocean initial conditions are of 00 UTC September 1. TR denotes the 3-dimensional observed ocean temperature and salinity trends estimated over 1960–2014 on 00 UTC September 1, using the PEODAS ocean reanalysis set. 99 forecasts were generated for El Niño with the present ocean mean state (pElNiño) and for El Niño with the warmer ocean mean state by the doubled observed trend (wElNiño), and 33 forecasts were generated for the present climatology (pClim) and the warmer climatology with the doubled observed trend (wClim).

The atmosphere and land component models of POAMA were initialised with 33 different conditions drawn from observed states during 1981–2013 in order to scramble atmosphere and land initial conditions within the observed range so to generate ensemble forecasts. The CO_2_ concentration was fixed to 345 ppm, which is the default value of POAMA retrospective forecast set, and the ozone concentration was prescribed by the observed monthly climatology^34^. Therefore, differences in the predicted atmospheric circulation in our experiments can be interpreted as responses to the differences in ocean forecasts in the experiments. We initialised all experiments on 1 September and limited our interest to the three month mean forecasts for October to December (OND) because ENSO and SAM are better correlated on a seasonal time scale than on shorter time scales and the three month mean observed correlation is maximum in OND^4^.

## Revisiting the Observed Relationship Between ENSO and SAM

Austral spring-summer SAM is influenced by both ENSO and Antarctic stratospheric vortex variation, the latter of which is considered to be a stronger driver^16^. SAM has also shown a strong positive trend from the 1980s to the late 1990s due to the anthropogenically-driven Antarctic ozone depletion^61^. To highlight the relationship of SAM with ENSO, which is largely an interannual variation, we de-trended the SAM index for the period of 1979–2016. Then, we regressed out the component of SAM related to the stratospheric polar vortex variation from the de-trended SAM index, using the SH stratosphere-troposphere coupled mode index^18^ as a predictor representing anomalous stratospheric conditions (i.e. de-trended residual SAM; see Methods for the climate indices used in the study).

**Figure 2:**
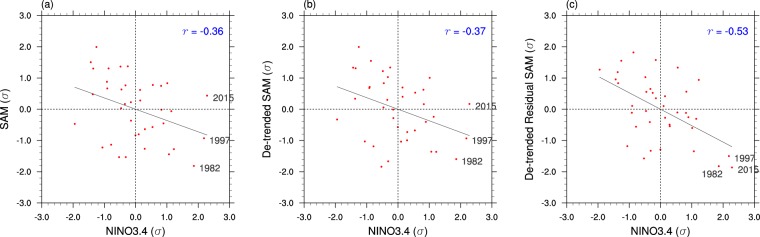
Observed ENSO and SAM relationship in 1979–2016. Scatter diagrams of Niño3.4 SST anomalies versus (**a**) SAM, (**b**) de-trended SAM, and (**c**) de-trended residual SAM after removing the influence of the Antarctic stratospheric vortex on SAM by using a linear regression. 1979–2016 October-November-December (OND) data are displayed in each diagram. The standardized Niño3.4 SST and SAM values are shown. The correlation of the SAM indices with Niño34 is displayed in the upper right of each panel. Correlation greater than |0.33| is statistically significant correlation at the 5% level (assuming 38 samples). The impact of de-trending the SAM index on the strength of the correlation with Niño3.4 SST is negligible as shown in (**b**).

Earlier studies^18,62^ reported no significant relationship between the Niño3.4 SST index, which represents eastern Pacific ENSO variability, and the Antarctic stratospheric vortex variation during the last 40 years, but SAM is significantly better correlated with Niño3.4 SST after the removal of the influence of the SH stratospheric polar vortex variation from the SAM index (*r* = −0.53, statistically significant at the 0.1% level (i.e. *p* < 0.001); Fig. 2c). The three extreme El Niño events of 1982, 1997 and 2015, which were accompanied by a substantial spread in the magnitude of raw SAM (e.g. SAM was neutral during the late spring of the strong 2015 El Niño), all exhibited strong low SAM after the removal of the stratospheric influence. This result supports the connection between ENSO and SAM in OND.

**Figure 3:**
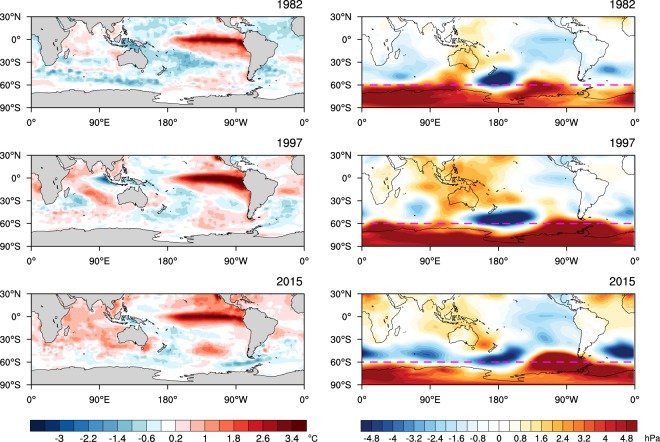
Observed SST and mean sea level pressure anomalies during three extreme El Niño years. Reynolds OI v2SST (SST; left panels) and ERA-Interim mean sea level pressure (MSLP) de-trended anomalies (right panels) for three strong El Niño years (1982, 1997 and 2015). The linear trend and the influence of the Antarctic polar vortex variability were removed from the MSLP anomalies by linear regression. On the right panels, the magenta dashed lines are drawn at 60°S as a reference for pressure dipole anomalies related to SAM. The colour shading interval for SST is 0.4 °C beginning at +/−0.2 °C and for MSLP is 0.4 hPa.

Figure 3 shows the SST anomalies of the three extreme El Niño events and de-trended residual MSLP anomalies of OND. Although there are differences in the spatial details and magnitudes of anomalies of SSTs, all three El Niño events are associated with low pressure anomalies equatorward of 60°S with a wavenumber 3 structure and high pressure anomalies poleward of 60°S, which typify low SAM. Strong stationary Rossby wave propagation from the Maritime Continent poleward and eastward to the Amundsen-Bellingshausen Seas is a distinctive teleconnection driven by El Niño, which is likely to further contribute to the amplitude of low SAM by placing a strong low pressure anomaly centre north of 60°S and a strong high pressure anomaly centre slightly south of 60°S. As noted in Fig. 2a, SAM was observed to be neutral despite the extreme strength of El Niño in OND 2015 because anomalous Antarctic stratospheric vortex strengthening^18^ and associated ozone depletion were acting to produce high SAM (https://ozonewatch.gsfc.nasa.gov/meteorology/figures/merra/ozone/toms_areas_2015_omi+merra.pdf). After removing this influence of the stratospheric polar vortex, the residual SAM is strongly negative with zonally symmetric pressure anomalies like those of 1982 and 1997 (Fig. 3 right panels). This implies that in 2015 the forcing of high SAM from a strengthening of the polar stratospheric vortex and associated depletion of Antarctic ozone may have completely countered the forcing of low SAM from El Niño in the OND season, thereby lowering the predictability of SAM and the associated surface climate.

**Figure 4:**
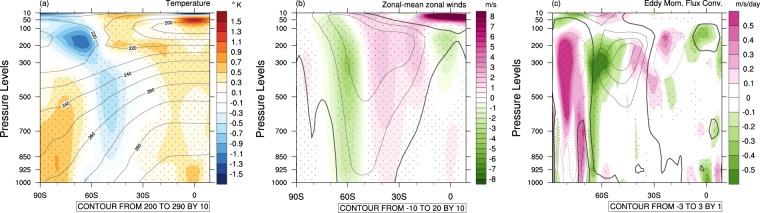
Observed composite anomalies of temperature, zonal wind and eddy momentum flux convergence of three extreme El Niños. (**a**) Zonal-mean temperature composites (colour shading) overlayed with the OND climatological temperature (contours). The colour shading interval is 0.2 °C, and the contour interval is 10 °C. (**b**) Same as (**a**) except zonal-mean zonal wind composites. The colour shading interval is 0.5 m/s, and the contour interval is 10 m/s. (**c**) Same as (**b**) except eddy momentum flux convergence composites. The colour shading interval is 0.1 m/s/day, and the contour interval is 1 m/s/day. Stippling indicates anomalies greater than 1 standard deviation (σ). The linear trend and the influence of the Antarctic polar vortex variability were removed from temperature, zonal wind and eddy momentum flux convergence data before forming the composites.

The promotion of low SAM by El Niño has previously been shown to result from intensification and equatorward contraction of the the Hadley circulation because the El Niño SST anomalies act to increase near equatorial diabatic heating (latent heat release as a result of moist atmospheric convection), which is flanked by enhanced diabatic cooling (longwave radiation to space as a result of enhanced subsidence and reduced latent heat release) in the subtropics (Fig. 4a). Thus, westerlies on the equatorward side of the climatological subtropical jet strengthen (Fig. 4b). This increase in westerly winds closer to the equator allows deeper penetration of extratropical baroclinic Rossby waves into the tropics^11,12^. As a result, eddy momentum flux divergence in the tropics shifts closer to the equator, and momentum flux convergence anomalously increases in the midlatitudes (30–40°S), while decreasing in the higher latitudes (50–70°S; Fig. 4c). Extratropical baroclinicity and the associated storm track thus shift equatorward, which is manifest as low SAM. These general features resulting from the eastern Pacific SST variations during El Niño, which were proposed in the literature, are confirmed by the composites of the three extreme El Niños in Fig. 4 and by the anomalies of the individual extreme El Niño in Supplementary Fig. S3.

## Experiment Results

### El Niño and SAM in the present climate

**Figure 5:**
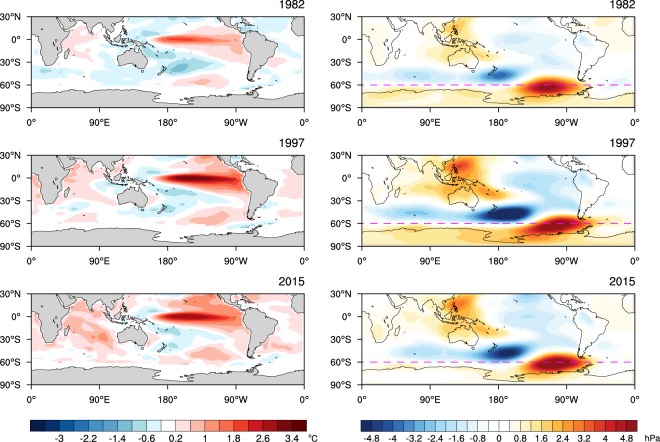
Forecast SST and MSLP for the three extreme El Niño years with the present mean state. 33-member ensemble mean forecast anomalies for present climate (pElNiño’ = pElNiño – pClim) of SSTs (left panels) and MSLP (right panels) for OND 1982, 1997 and 2015. The colour shading interval for SST is 0.4 °C beginning at +/−0.2 °C and for MSLP is 0.4 hPa. On the right panels, the magenta dashed lines are drawn at 60°S as a reference for pressure dipole anomalies related to SAM.

Figure 5 displays 33-member ensemble mean anomalies of SSTs and MSLP averaged during OND for the present climate 1982, 1997 and 2015 El Niños. Anomalies are formed relative to the present climate as pElNiño’ = pElNiño-pClim. The strong El Niño events are skillfully predicted at this short lead time of 1 month, although the amplitude of 1982 El Niño is substantially underpredicted (by ~0.7 °C; Supplementary Fig. S4). In all three El Niño cases, overall patterns of forecast MSLP anomalies feature low SAM with low pressure anomalies being dominant equatorward of 60°S and high pressure anomalies over the polar cap, and a stationary Rossby wave train extending from the Maritime Continent poleward and eastward to the south eastern Pacific, which are consistent with the observed anomalies shown in Fig. 3. However, a strong low-pressure center observed over the southern Atlantic Ocean is missed in all three of the simulated El Niño cases (Fig. 5 right panels). Reproducibility of low SAM in pElNiño’, which is initialised with realistic ocean conditions but random atmosphere and land conditions, confirms that strong El Niño can be an important forcing of low SAM, and the polarity of SAM would have been more negative during El Niño of 2015 if the Antarctic polar vortex were not significantly stronger than usual.

### Warmer climate mean state

**Figure 6:**
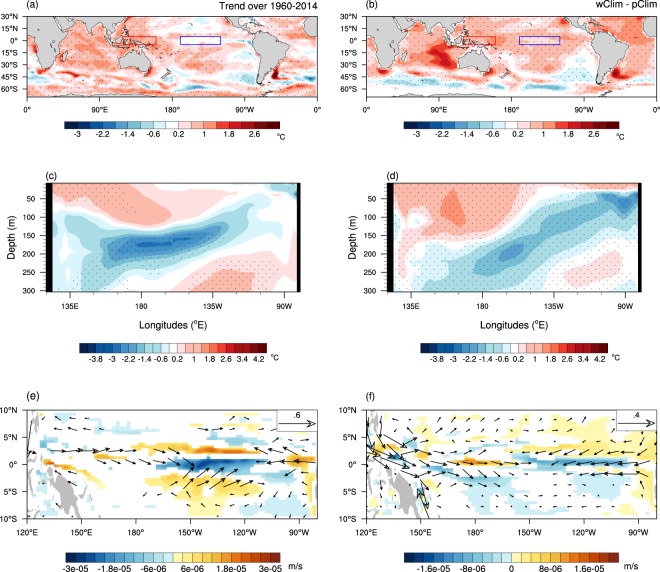
Simulated mean state changes in SST and Pacific equatorial subsurface temperature and tropical upper ocean circulation. Comparisons between (left panels) the observed trends and (right panels) the mean state changes simulated by POAMA (wClim-pClim) for OND. (**a**,**b**) SSTs, (**c**,**d**) equatorial Pacific temperatures in the upper 300 m, (**e**,**f**) vertical velocities at the bottom of the mixed layer (45 m) (colour shading) overlayed with the horizontal velocities of currents averaged in the mixed layer (vectors). SSTs averaged over the red and blue boxes in (**a**) and (**b**) were used to estimate the zonal SST gradients^40^. The colour shading interval is 0.4 °C beginning at +/−0.2 °C for temperatures in (**a**–**d**) and 3.0*10^−6^ and 2.0*10^−6^ m/s for vertical velocity in (**e**) and (**f**), respectively. The size of the reference vector is 0.6 and 0.4 m/s in (**e**) and (**f**), respectively. Statistical significance at the 5% level is stippled in (**a**–**d)**, and in (**e**) and (**f**) the zonal and vertical velocity trends and changes significant at the 5% level are displayed (see Methods for details of statistical significance tests).

For the results of the forecasts of extreme El Niño in the idealised future warmer climate (wElNiño) to be scientifically reliable and attributable to the change in the mean state of the initial conditions, key features of the observed ocean temperature trends added in the initial conditions and resultant ocean circulation changes should be faithfully maintained through the October to December verification period. This is demonstrated by comparing mean differences in SSTs, equatorial Pacific upper ocean temperatures and tropical Pacific mixed layer circulations between wClim and pClim to the observed trends in OND over 1960–2014 in Fig. 6. We note that although we added doubled the observed ocean temperature trends to the climatological ocean initial conditions for wClim, the magnitudes of the ocean surface and subsurface warming over the following four months are more or less comparable to the magnitudes of the observed trends of the 55 years of 1960–2014. This loss of amplitude in the mean state trend may be partly due to the scrambled atmosphere initial conditions and their damping effect until the atmosphere and the ocean are brought to a balance in the first month of forecasts^24^, or the lack of changing the greenhouse gas forcing in the warmer climate runs. Nevertheless, it is encouraging to see the overall similarities between the warmer minus present climate differences and the observed trends for the spatial structures of SSTs, equatorial Pacific subsurface temperatures and tropical Pacific mixed layer ocean circulations. For instance, the pattern correlation between Figs. 6a,b is 0.55 (*p* < 0.001 with 5760 grid values), and the enhanced zonal SST gradient between the tropical western Pacific (120–160°E, 5°S-5°N)^40^ and eastern Pacific (Niño3.4 region) is maintained in OND forecasts in wClim minus pClim although the gradient is simulated with only about one third of the observed magnitude (0.3 °C compared to 0.9 °C). The observed reduction of upwelling into the mixed layer (averaged 0–45 m depth) in the equatorial eastern Pacific is also faithfully captured by wClim forecasts (Figs. 6e,f). On the other hand, forecasts of SSTs and subsurface temperatures are significantly different from the observed trends in the far eastern Pacific east of 120°W, which should be borne in mind when we diagnose below how the change in the mean state acts to change El Niño growth.

**Figure 7:**
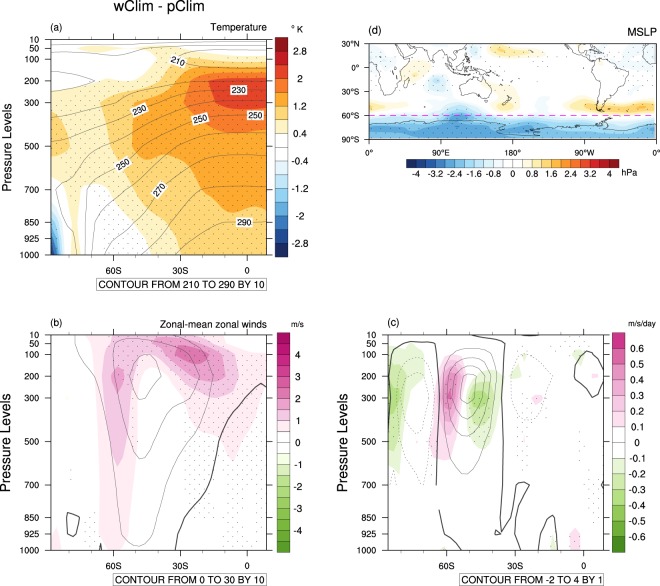
Simulated changes in air temperature, zonal-mean zonal wind, eddy momentum flux convergence and MSLP due to the ocean mean state difference. 33-member ensemble mean differences of temperature, zonal wind and eddy momentum flux convergence and MSLP between the warmer climate (wClim) and the present climate (pClim). (**a**) Zonal-mean temperature difference (colour shading) overlayed with the climatological temperatures of pClim (contours). The colour shading interval is 0. 4 °C, and the contour interval is 10 °C. (**b**) Same as (**a**) except zonal-mean zonal wind difference. The colour shading interval is 0.5 m/s, and the contour interval is 10 m/s. (**c**) Same as (**b**) except eddy momentum flux convergence difference. The colour shading interval is 0.1 m/s/day, and the contour interval is 1 m/s/day. Eddy momentum flux convergence differences obtained from model interpolated data over the Antarctic continent are masked. (**d**) MSLP difference. The colour shading interval is 0.4 hPa. The magenta dashed line in (**d**) is drawn at 60°S as a reference for pressure dipole anomalies related to SAM. Stippling indicates statistical significance of the difference of the means of the two simulations at the 5% level.

The atmospheric response to the warmer climate (Fig. 7a) shows more intense warming in the upper troposphere in the tropics than in the higher latitudes of the SH, steepening the meridional temperature gradient between the tropics and the south pole. This change results in a poleward shift of the eddy-driven jet with anomalously increased eddy momentum flux convergence around 60°S but decreased eddy momentum flux convergence around 45°S (Figs. 7b,c). The associated MSLP change projects onto high SAM (standardized SAM index = 0.9), although the high-pressure anomalies in the midlatitudes are not as annular as that of conventional SAM (Fig. 7d). These atmospheric changes are similar to those projected by climate models in response to increasing greenhouse gases^63^ despite the contrasting tropical Pacific zonal SST gradients between wClim and climate change simulations. The similarity in the atmospheric responses despite differences in the degree of warming in the tropical eastern SSTs is because equatorial waves quickly spread anomalous temperature change across the entire tropics^59^.

### El Niño on the warmer ocean mean state

We now turn to the warmer climate El Niño compared to the present climate El Niño. Because forecasts of the SST and MSLP patterns of the three El Niño years in the present climate appear to be all very similar to one another, we will present results of a grand ensemble of 99 members by combining the anomalies for the 1982, 1997 and 2015 simulations.

**Figure 8:**
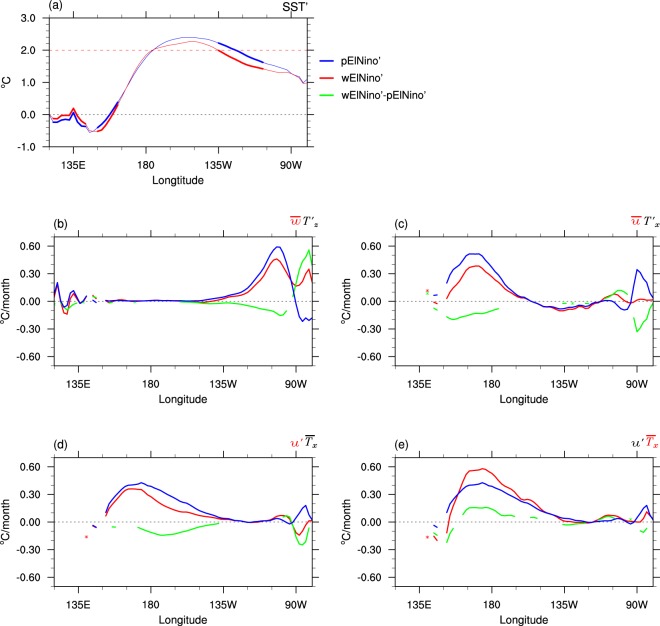
Changes in extreme El Niño and its ocean mixed layer heat budget due to the ocean mean state difference. Equatorial anomalies of present climate El Niño (pElNiño’, blue curves) and warmer climate El Niño (wElNiño’, red curves) for (**a**) SSTs and (**b**–**e**) the ocean mixed layer heat advective terms. Anomalies are computed over 5°S-5°N. Thick solid curves in (**a**) and green curves in (**b**–**e)** indicate where the difference between pElNiño’ and wElNiño’ is significant at the 5% level, using the two sets of 99 member forecasts. The displayed ocean mixed layer heat advective terms are (**b**) thermocline feedback by the mean ocean upwelling at 45 m for pClim and wClim ($\bar{w}$) acting on the anomalous vertical temperature gradients (*T*′*z*) of pElNiño’; (**c**) zonal advective feedback by the mean zonal currents (averaged in the mixed layer) of pClim and wClim ($\bar{u}$) acting on the anomalous zonal temperature gradients (*T*′*x*) of pElNiño’; (**d**) zonal advective feedback by the anomalous zonal currents of pElNiño’ and wElNi*ñ*o’ (*u*′) acting on the mean zonal temperature gradients of pClim $({\bar{T}}_{x})$; and (**e)** zonal advective feedback by the anomalous zonal currents of pElNiño’ (*u*′) acting on the mean zonal temperature gradients of pClim wClim (${\bar{T}}_{x}$). Plots of all the individual ocean heat advection terms can be found in Supplementary Figs. S5–7.

When El Niño of the same extreme strength occurs on the “La Niña-like” warmer ocean mean state (wElNiño’ = wElNiño-wClim), its strength is simulated to be weaker than that in the current climate (pElNiño’ = pElNiño-pClim) over the tropical eastern Pacific (*p* < 0.05, see Methods for statistical significance calculation; Fig. 8a, Supplementary Fig. S4). Therefore, the maximum warming of El Niño in the warmer mean state appears to be more confined to the central Pacific, giving it some central Pacific flavour. Analysis of advective feedback terms in the ocean mixed layer heat budget (averaged over 0–45 m, 5°S-5°N; see Methods) suggests that the reduced mean upwelling in the eastern Pacific in the warmer ocean mean state (Fig. 6f) is partly responsible for the weaker growth of eastern Pacific SST anomalies in the warmer climate (Fig. 8b). Across the central to western Pacific, the reduced strength of mean westward surface currents in the warmer ocean mean state (Fig. 6f) also contributes to weaker growth of warm anomalies over and west of the dateline (Fig. 8c), while weaker anomalous eastward currents during El Niño in the warmer climate contributes to weaker warming over the central Pacific (Fig. 8d). The cause of the weaker anomalous eastward currents during El Niño in the warmer climate is addressed below. In contrast, the enhanced mean zonal temperature gradient of the warmer ocean mean state over the equatorial Pacific (e.g. Figs. 6b,d) positively contributes to the growth of positive SSTs during El Niño over the central to western Pacific (Fig. 8e), thereby compensating for some of the negative contributions caused by the weakening of the mean westward zonal currents and the weaker El Niño-generated anomalous eastward currents there.

**Figure 9:**
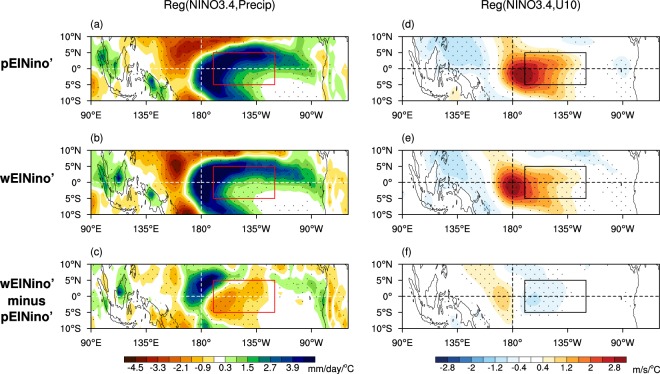
Changes in rainfall and surface zonal winds associated with extreme El Niño due to the ocean mean state difference. Estimates of air-sea coupling during present climate El Niño (pElNiño’; panels) and warmer climate El Niño (wElNiño’; middle panels). (**a**,**b**) Regression of rainfall anomalies onto the Niño3.4 SST anomalies (red box) of OND forecasts, using the 99 member ensemble. (**c**) Difference of regression coefficients between (**a**,**b**). The colour shading interval is 0.6 mm/day per 1 °C Niño3.4 SST anomaly beginning at +/−0.3 mm/day/°C. (**d**–**f**) Same as (**a**–**c**) except regression of the 10-m zonal wind anomalies onto the Niño3.4 SST anomalies (black box). The colour shading interval is 0.4 m/s per 1 °C Niño3.4 SST anomaly. Stippling indicates that regression coefficients in (**a**,**b**,**d**,**e**) and the differences of regression coefficients between pElNiño’ and wElNiño’ in (**c**) and (**f**) are statistically significant at the 5% level.

The cause of the weaker El Niño-generated anomalies in the waremer mean state can be traced to westward shifts of the maximum rainfall and wind responses to the El Niño SST anomalies while reducing their responses in the tropical eastern Pacific. These changes are quantified by the regressions of rainfall and 10-m zonal wind anomalies onto the Niño3.4 SST anomalies during OND (all 99 forecast members are used for the regression calculation). Figures 9a–c show that El Niño-driven rainfall in the warmer climate is shifted westward relative to that in the present climate, and over the Niño3.4 region the rainfall anomaly is weaker (*p* < 0.05). This likely occurs due to the cooler equatorial mean SSTs (Fig. 6b) and weaker El Niño SST anomalies caused by the reduced thermocline feedback in the eastern Pacific (Figs. 8a,b), which makes a SST anomaly less efficient at driving a rainfall/wind response^60,64,65^. In contrast, there is an increased rainfall response over and west of the dateline during wElNiño’ compared to pElNiño’ likely boosted by the warmer SST mean state of the tropical central to western Pacific, shifting the location of the maximum rainfall associated with the Niño3.4 SSTs about ~10° westward (*p* < 0.05). Likewise, SST anomalies over the Niño3.4 region induce weaker westerly response over the eastern Pacific but stronger westerly response west of the dateline with the warmer mean state compared to the present mean state (*p* < 0.05; Fig. 9d–f). Together these westward shifts of the zonal wind and rainfall responses during El Niño on the warmer mean state feed back into producing a weaker El Niño in the eastern Pacific.

Interestingly, these 1) weakened thermocline feedback and 2)air-sea coupling strength and resultant weakened El Niño with its maximum SST warming and rainfall response concentrated in the central Pacific are consistent with what has been diagnosed to have occurred during the early 2000s as compared to the 1980s and 1990s and is thought to reflect the shift to the cold phase of the IPO^60,64–67^. The cold phase of the IPO is also characterised by an enhanced zonal SST gradient across the tropical Pacific and shallower thermocline in the equatorial eastern Pacific, but there are many differences between the cold phase of the IPO and our warmer ocean mean state (Supplementary Fig. S1). Hence, the consistency in the changes in the ocean and atmosphere feedback and El Niño properties between our warmer ocean mean state and the cold phase of IPO highlight the role of the equatorial Pacific mean state in modulating the amplitude of El Niño and the longitude of maximum warming.

### El Niño and SAM on the warmer ocean mean state

**Figure 10:**
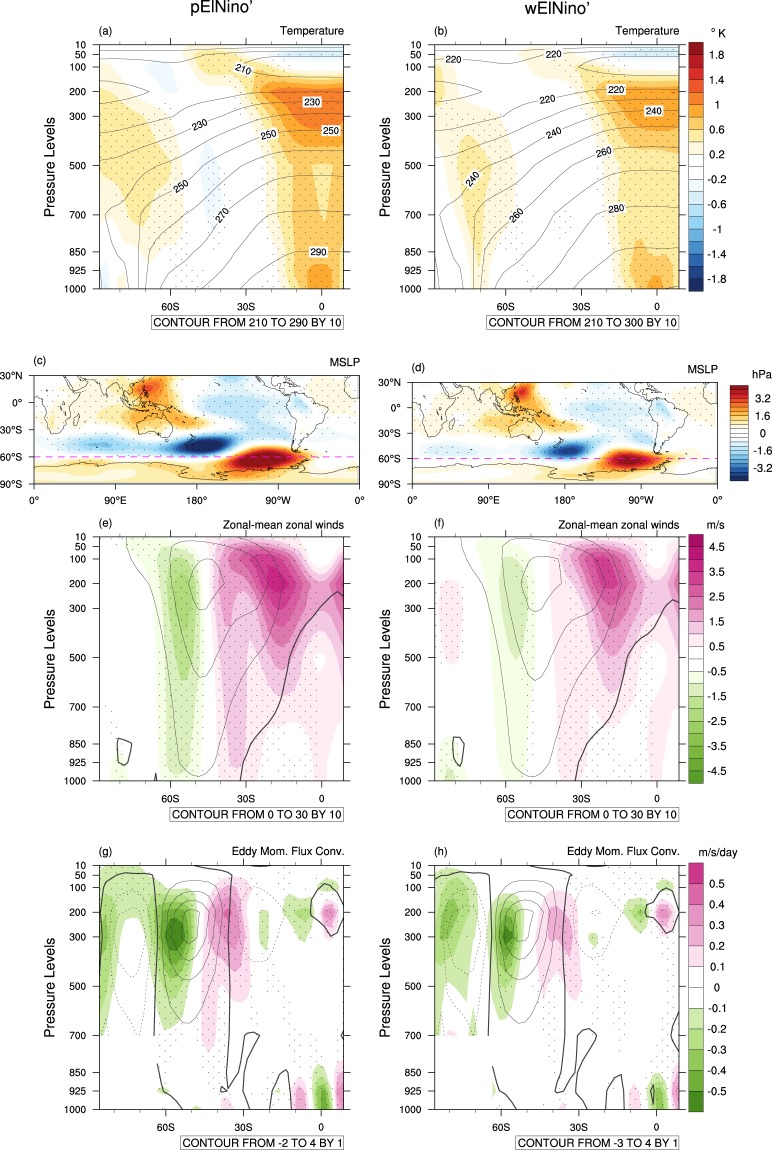
Changes in air temperature, MSLP, zonal-mean zonal wind and eddy momentum flux convergence associated with extreme El Niño due to the ocean mean state difference. 99-member ensemble mean anomalies of temperatures, MSLP, and upper tropospheric zonal winds and eddy momentum flux convergence for pElNiño’ and wElNiño’. (**a**) Temperature anomalies (pElNiño’; colour shading) overlaid by its climatology (pClim; contours). (**b**) Same as (**a**) expcept wElNiño’ and wClim. The colour shading interval is 0.2 °C, and the contour interval is 10 °C. (**c**,**d**) Same as (**a**,**b**) except MSLP anomalies. The magenta dashed lines are drawn at 60°S as a reference for pressure dipole anomalies related to SAM. The colour shading interval is 0.4 hPa. (**e,f**) Same as (**a,b**) except zonal-mean zonal wind anomalies. The colour shading interval is 0.5 m/s, and the contour interval is 10 m/s. (**g,h**) Same as (**a,b**) except anomalies of eddy momentum flux convergence. The colour shading interval is 0.1 m/s/day, and the contour interval is 1 m/s/day. Eddy momentum flux convergence anomalies obtained from model interpolated data over the Antarctic continent are masked. Stippling indicates that the differences between pElNiño’s and wElNiño’ are statistically different at the 5% level.

Although El Niño weakens on the warmer ocean mean state, it still promotes low SAM. However, the El Niño-low SAM connection is substantially weaker in the warmer climate compared to the current climate as judged by standardized SAM being −0.7 as derived from the pressure anomalies of Fig. 10d compared to standardized SAM being −2.1 as derived from those of Fig. 10c (this weakening of the SAM strength is statistically significant at the 1% level). The Rossby wave train emanating from the Maritime Continent is also weaker in the warmer climate. This weakening of teleconnection is likely due to both weakening of El Niño and weakening of the tropical convective heating response to El Niño on the warmer mean state. As shown in Figs. 10a,b, wElNiño’ induces less intense warming over the tropical upper troposphere, and therefore, the meridional temperature gradient between the tropics and the SH midlatitudes is not as steep as that caused by pElNiño’, resulting in weakened westerly anomalies associated with wElNiño’ compared to those with pElNiño’s (Figs. 10e,f). This reduced change in the mean winds in the upper troposphere leads to reduced changes in eddy momentum flux convergence anomalies in the subtropics to the high latitudes of the SH (Fig. 10g,h); which feed less of anomalous momentum back to the mean flow, resulting in weaker dipole wind anomalies in the SH extratropics, leading to weaker low SAM. The strengths of the extratropical zonal-mean zonal wind dipole and the eddy momentum flux convergence dipole in the upper troposphere are statistically significantly different between pElNiño’ and wElNiño’ at the 5% level (Supplementary Fig. S8). Consequently, predictability of SAM in OND would be substantially reduced during El Niño under our scenario of continuation of the observed ocean temperature trends to the future.

## Concluding Remarks

This study was motivated by earlier research findings that ENSO is an important source of predictability of SH extratropical climate through its connection to SAM during austral spring and summer; yet this ENSO-SAM relationship is not skillfully simulated by climate models, therefore making it hard to foresee any possible change in this relationship in a future warmer climate. Furthermore, climate models have not been able to reach a strong consensus on how ENSO amplitude will change^43^, which would be a key determinant in predictability of extratropical climate via its teleconnections. The strength of ENSO and its spatial characteristics influence and are influenced by the tropical ocean mean state, especially the tropical Pacific Ocean mean state^60,66^. There is still vigorous debate, though, about whether the response of the tropical Pacific to global warming will be more “La Niña-like” with greater warming over the tropical western Pacific than over the eastern Pacific, which is what has been observed over the past 60 years^39^, or more “El Niño-like” with the opposite pattern of warming, which is projected by the majority of the CMIP5 models^47^.

In this study, we limited our focus to El Niño and attempted to address this question: How will extreme El Niño and its relationship with SAM change in a warmer climate if the “La Niña-like” ocean temperature trends that have occurred over the past 60 years continue into the future? To do so, we conducted a series of forecast sensitivity experiments for three extreme El Niños (1982, 1997 and 2015). The forecast simulations were initialised on 1 September using the present ocean mean state and using a hypothetical future ocean mean state that was created by addition of the doubled observed ocean temperature trends over the past 60 years (we have referred to this as the warmer ocean mean state). Present and warmer climatological forecasts were also produced with the climatological conditions of 1 September of 1981–2013 without and with the addition of the doubled observed ocean temperature trends, respectively, as reference forecast sets.

Statistical analysis of observations and the forecast experiments confirmed that extreme El Niño is a key driver of strong low SAM in the October to December season. The observed El Niño-low SAM relationship is robust once we remove the influence of the Antarctic stratospheric vortex on the SAM by regressing it out in the observational analysis. This conclusion is also supported by the forecast experiments that use scrambled atmospheric initial conditions so that the promotion of low SAM during the El Niño forecasts can be attributed to the El Niño forcing.

The El Niño experiments with the present and the hypothetical warmer ocean mean states revealed that extreme El Niño is likely to lose some strength particularly over the tropical eastern Pacific as a result of reduced mean upper ocean upwelling on the warmer ocean mean state which causes a reduction in the thermocline feedback. In our experiments, the increased zonal temperature gradient of the observed La Niña-like upper ocean mean state change contributes to anomalous SST warming associated with extreme El Niño, as highlighted by the recent study of Wang *et al*.^68^ However, this enhanced SST growth appears to be limited to the central and western Pacific^78^ and is offset by weaker anomaly growth caused by the reduced strength of mean westward surface currents in the same region. The air-sea coupling strength appears to also significantly weaken in the eastern Pacific due to the cooler equatorial mean SST and weaker SST anomalies associated El Niño and shift westward in the warmer climate, further contributing to the weakening of extreme El Niño in the eastern Pacific. This delicate balance of processes in our model framework tips the scale in favor of weaker extreme El Niño events in a warmer world with the continuation of the observed long-term ocean temperature trends.

The weakened El Niño in the warmer climate appears to result in a significant weakening of the low SAM response. Our present climate El Niño experiment confirmed the findings of earlier studies^10–12^ that tropical upper tropospheric warming caused by El Niño increases westerlies on the equatorward side of the SH subtropical jet, shifting the critical latitude equatorward. This shift induces an equatorward shift of the momentum flux convergence-divergence dipole, resulting in low SAM. This chain of processes appears to continue to operate in the warmer climate but with significantly weaker strength of zonal wind, eddy momentum transport and pressure anomalies because El Niño itself becomes weaker and the tropical convective heating response to El Niño becomes weaker as well. Therefore, if the ocean temperature trends that have been observed over the past 60 years continue into the future, predictability of low SAM and its associated impacts on SH surface climate during extreme El Niño is likely to be substantially reduced. A previous study^60^ indicated that La Niña may also be expected to weaken in response to a “La Niña-like” temperature trend, but a further study is required to assess whether there will be a similar reduction in predictability of high SAM. An idealised experiment like ours but to examine the ENSO-SAM relationship with El Nino-like warming trends in the tropical Pacific, as suggested by most CMIP models, would also be valuable in understanding the mechanisms of the resultant changes in ENSO and SAM.

## Methods

### Data for observational analyses

We used reanalysis data of the European Centre for Medium-Range Weather Forecasts Interim project (ERA-Interim)^69^ and SST analyses of Hurrell *et al*.^70^ and Reynolds OI v2^71^ for the period of 1979–2016. Anomalies were computed against the base period of 1981–2013 for comparisons with POAMA experimental forecasts. The 55 year observed temperature and upper ocean circulation trends were calculated with POAMA ocean data assimilation system reanalysis data (PEODAS)^72^ for the period 1960–2014.

### Climate indices

The SAM index was obtained by following Gong and Wang’s definition^73^, which is the difference between normalised zonally averaged MSLP anomalies at 40°S and 65°S. The strength of extreme El Niños was determined by the Niño3.4 index, which was obtained by averaging SSTs over the domain of 5°S-5°N, 190–240°E. The stratosphere-troposphere (S-T) coupled mode index was obtained following the method of Lim *et al*.^18^ by applying height-time domain EOF analysis to anomalies (seasonal cycle removed) of monthly mean zonal-mean zonal winds averaged over 55–65°S. The input data to the EOF was ordered from April to March each year for pressure level data extending from 1000 to 1 hPa. The resultant 1^st^ principal component time series consists of one value each year and depicts the year-to-year variations of the SH spring polar vortex strength and its downward coupling. To obtain the de-trended residual SAM independent of the influence of the Antarctic stratospheric polar vortex, we removed from the SAM index the components linearly related to time and the S-T coupled mode index variability.

### Model and initial conditions for forecast sensitivity experiments

For the model experiments, we used the Bureau of Meteorology’s atmosphere-ocean fully coupled dynamical seasonal climate forecast system, POAMA version a^34^. Its atmosphere and ocean component models are the Bureau’s Atmosphere Model version 3 (T47/L17)^74^, and the Australian Community Ocean Model version 2 (2° longitude by 0.5–1° latitude from the tropics to the pole)^75^, which are coupled by OASIS^76^ coupler.

High quality observed atmosphere, land and ocean conditions are generated from the Bureau of Meteorology’s atmosphere and land initialisation scheme (ALI)^77^ and PEODAS, respectively.

### Statistical significance tests

Statistical significance of the observed trends in Figs 1 and 6 was assessed by a two-sided Student t-test with 55 samples (i.e. data of 1960–2014), using the incomplete beta function available in NCAR Command Language. In Fig. 4, 1 standard deviation threshold was used to indicate if air temperature, zonal wind and eddy momentum flux convergence anomalies associated with extreme El Niño are significantly different from the climatological conditions in the ERA-Interim reanalysis set. A two-sided Student t-test was used to estimate the statistical significance on the difference of the two means (Figs 8, 9a,b,d,e, 10) and of the two regression coefficients (Fig. 9c,f) with 99 samples for p’ElNiño vs. w’ElNiño and and the difference of the two means with 33 samples for pClim vs wClim (Figs 6b,d,f and 7).

### Ocean mixed layer heat advection analysis

Based on the ocean mixed layer heat budget and heat advection analyses of Zhao *et al*.^60^ and Abellan *et al*.^78^, we computed contributions of advective feedback terms to the change of El Niño growth as follows:

1 $$\frac{\partial T}{\partial t}\approx -\,{\boldsymbol{u}}\nabla T$$

2 $${\boldsymbol{u}}\nabla T=\bar{u}T{^{\prime} }_{x}+u^{\prime} {\bar{T}}_{x}+u^{\prime} T{^{\prime} }_{x}+\bar{v}T{^{\prime} }_{y}+v^{\prime} {\bar{T}}_{y}+v^{\prime} T{^{\prime} }_{y}+\bar{w}T{^{\prime} }_{z}+w^{\prime} {\bar{T}}_{z}+w^{\prime} T{^{\prime} }_{z}$$

where ***u*** denotes zonal, meridional and vertical velocities, and *T*_*x*_, *T*_*y*_ and *T*_*z*_ indicate zonal (*x*), meridional (*y*) and vertical (*z*) temperature gradients, respectively. Overbar and prime signs denote a temporal mean and a departure from the mean, respectively.

From Eq. (2), we further limited our interest to linear terms to tease out the contributions from the mean state changes vs the anomalous changes to changes in the growth of El Niño. To estimate the contributions of the mean state changes, we used the mean state terms $\bar{u}$, $\bar{v}$, $\bar{w}\,$, ${\bar{T}}_{x}$, ${\bar{T}}_{y}$, and ${\bar{T}}_{z}$ from the two different climatologies, pClim and wClim, together with the anomalies from the present climate *u*′, *v*′, *w*′, *T*′_*x*_, *T*′_*y*_ and *T*′_*z*_. Then, to estimate the contributions of the anomalous changes in El Niño, we used the anomalies *u*′, *v*′, *w*′, *T*′_*x*_, *T*′_*y*_ and *T*′_*z*_ of El Niño for the two different mean states, wElNiño’ and pElNiño’, together with the mean state $u^{\prime}$, $v^{\prime}$, $w^{\prime}$, ${T^{\prime} }_{x}$, ${T^{\prime} }_{y}$, and ${T^{\prime} }_{z}$ from the present climatology (pClim).

Figure 8 and Supplementary Figs. S5–7 show that the most important term that explains the reduction of El Niño strength in the tropical eastern Pacific is the thermocline feedback change by the reduced mean upwelling on the warmer ocean, $\bar{w}{{T}^{^{\prime} }}_{z}$, in Eq. (2). The reduced mean westward currents of the warmer mean state in $\bar{u}T{\text{'}}_{x}\,\,$and the reduced anomalous eastward current of warmer climate El Niño in $u^{\prime} {\bar{T}}_{x}\,\,$also suggest some contributions to the reduction of SST warming associated with warmer climate El Niño over the tropical central Pacific. However, as the enhanced zonal temperature gradient of the warmer mean state in $u^{\prime} {\bar{T}}_{x}\,\,$somewhat compensates the temperature reduction, an overall contribution from the zonal advective feedback change to the change in the SSTs of warmer climate El Niño seems small.

## Supplementary information

Dataset 1
