# Publisher Correction: Continuation of tropical Pacific Ocean temperature trend may weaken extreme El Niño and its linkage to the Southern Annular Mode

**DOI:** 10.1038/s41598-020-58288-w

**Published:** 2020-02-04

**Authors:** Eun-Pa Lim, Harry H. Hendon, Pandora Hope, Christine Chung, Francois Delage, Michael J. Mcphaden

**Affiliations:** 1 Bureau of Meteorology, Melbourne, VIC 3000 Australia; 2 Pacific Marine Environmental Laboratory, National Oceanic and Atmospheric Administration, Seattle, WA USA

Correction to: *Scientific Reports* 10.1038/s41598-019-53371-3, published online 19 November 2019

The original version of this Article contained a typographical error in the ‘Concluding Remarks’ section, where Reference 65 was inadvertently cited as Reference 78.

As a result,

“However, this enhanced SST growth appears to be limited to the central and western Pacific^78^ and is offset by weaker anomaly growth caused by the reduced strength of mean westward surface currents in the same region. The air-sea coupling strength appears to also significantly weaken in the eastern Pacific due to the cooler equatorial mean SST and weaker SST anomalies associated El Niño and shift westward in the warmer climate, further contributing to the weakening of extreme El Niño in the eastern Pacific.”

now reads:

“However, this enhanced SST growth appears to be limited to the central and western Pacific^65^ and is offset by weaker anomaly growth caused by the reduced strength of mean westward surface currents in the same region. The air-sea coupling strength appears to also significantly weaken in the eastern Pacific due to the cooler equatorial mean SST and weaker SST anomalies associated El Niño and shift westward in the warmer climate, further contributing to the weakening of extreme El Niño in the eastern Pacific.”

In addition, the HTML version of this Article contained a typographical error in the ‘Concluding Remarks’ section.

“In our experiments, the increased zonal temperature gradient of the observed La Niña-like upper ocean mean state change contributes to anomalous SST warming associated with extreme El Niño, as highlighted by the recent study of Wang *et al*.68”

now reads:

“In our experiments, the increased zonal temperature gradient of the observed La Niña-like upper ocean mean state change contributes to anomalous SST warming associated with extreme El Niño, as highlighted by the recent study of Wang *et al*.^68^”

Finally, the Article contained an error in the References where the link for Reference 65 was inadvertently omitted.

These errors have now been corrected in the HTML and PDF versions of this Article.

