# Supplementary material for: Continuation of tropical Pacific Ocean temperature trend may weaken extreme El Niño and its linkage to the Southern Annular Mode
Source: Sci Rep. 2019 Nov 19;9:17044. doi: 10.1038/s41598-019-53371-3 (PMC6864090; doi:10.1038/s41598-019-53371-3)
Supplement: Supplementary file 1 — Dataset 1 [file 41598_2019_53371_MOESM1_ESM.pdf]

Supplementary Information

Continuation of tropical Pacific Ocean temperature trend may weaken the linkage of Southern Annular Mode and extreme El Niño

Eun-Pa Lim[1], Harry H. Hendon[1], Pandora Hope[1], Christine Chung[1], Francois Delage[1] and Michael J. McPhaden[2]

[1]Bureau of Meteorology, Melbourne, VIC 3000, Australia

[2]Pacific Marine Environmental Laboratory, National Oceanic and Atmospheric Administration, Seattle, WA, USA

Corresponding author: Eun-Pa Lim

e.lim@bom.gov.au

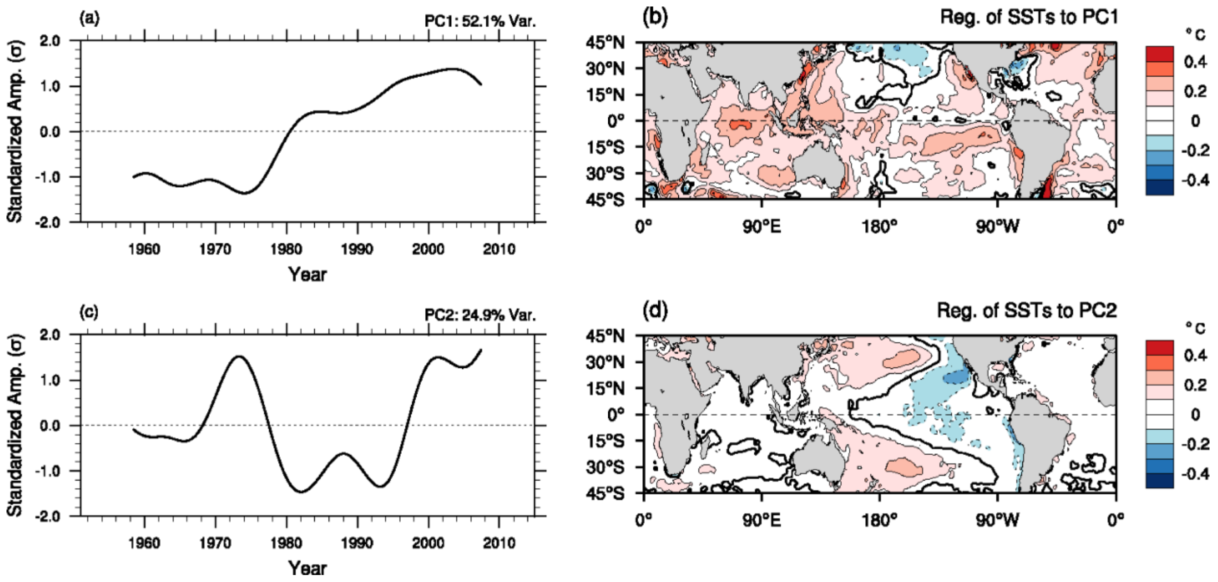

Supplementary Figures S1| Dominant modes of decadal variability of Pacific SSTs. First and second empirical orthogonal function[1] (EOF) modes of 15-yr low-pass filtered SST variability over the Pacific domain of 30°S-30°N, 120-300°E for the period 1951-2014 using Hurrell et al SST analyses[2]. (a), (c) 1st and 2nd principal component time series (PC). (b), (d) Regression patterns of SST anomalies on the standardized 1st and 2nd PCs, respectively. The colour shading interval is 0.1°C.

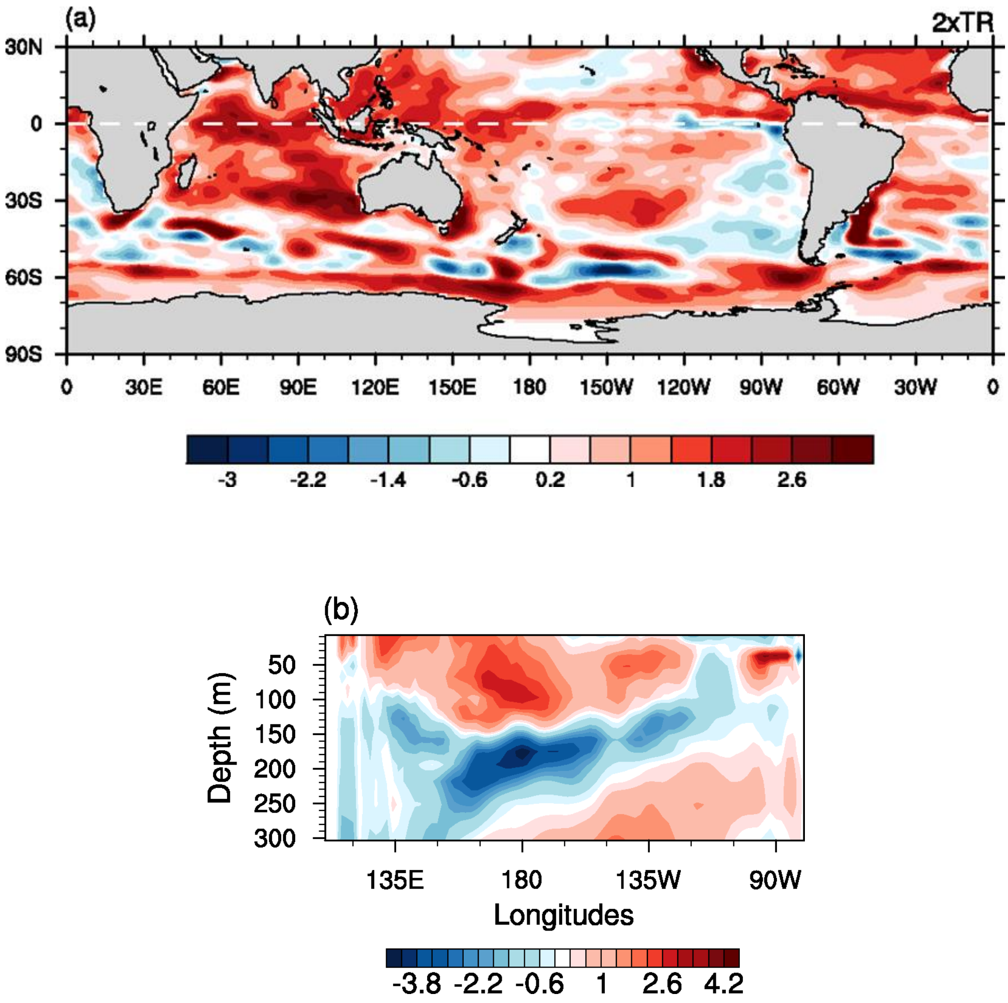

Supplementary Figure S2| Doubled magnitudes of the observed temperature trends. Doubled (a) SST and (b) equatorial Pacific ocean subsurface temperature trends. The doubled 3-dimensional ocean temperature and salinity trends were added to the ocean conditions for 1982, 1997 and 2015 El Nino forecasts (wElNino) and climatological forecasts (wClim). The trends were estimated over 1960-2014 at 00 UTC 1 September, using PEODAS ocean reanalysis data[3].

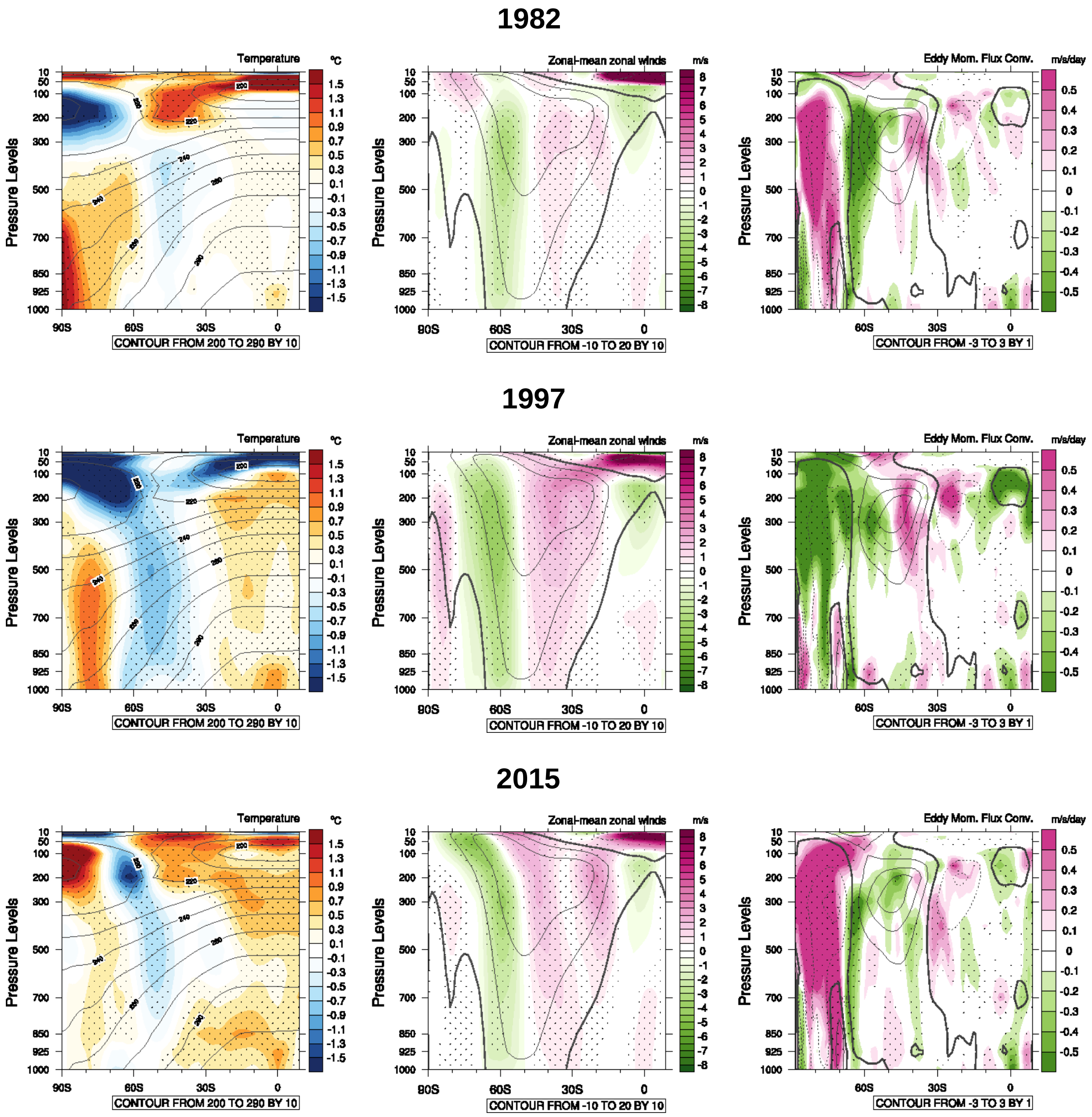

Supplementary Figure S3| Anomalous atmospheric conditions of the three El Nino events of 1982, 1997 and 2015 in the October-November-December season. (Left panels) Zonal-mean temperature anomalies (shading) overlayed with the climatology (contours). The colour shading interval is 0.2°C, and the contour interval is 10°C. (Middle panels) Zonal-mean zonal wind anomalies overlayed with the climatology. The colour shading interval is 0.5 m/s, and the contour interval is 10 m/s. (Right panels) Eddy momentum flux convergence anomalies overlayed with the climatology. The colour shading interval is 0.1 m/s/day, and the contour interval is 1 m/s/day. Stippling indicates anomalies greater than 1 standard deviation (σ). The linear trend and the influence of the Antarctic polar vortex variability were removed from temperature, zonal winds and eddy momentum

flux convergence data before forming the composites. The three observed extreme El Nino events have some common atmospheric responses such as the tropical warming-midlatiude cooling-high latitude warming pattern in the Southern Hemisphere (SH) (left panels); strengthening of the subtropical jet with a dipole of the extratropical wind anomalies (middle panels), indicating an equatorward shift of the eddy-driven jet; and a decrease of eddy momentum flux convergence deep in the tropics with a dipole of the eddy momentum flux convergence anomalies in the upper troposphere (right panels).

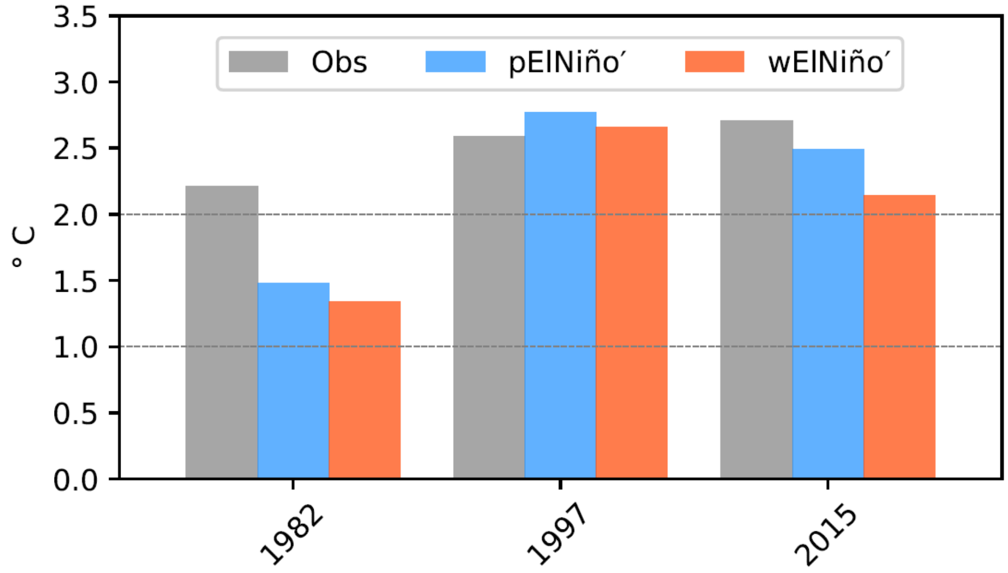

Supplementary Figure S4| Observed and forecast extreme El Nino of 1982, 1997 and 2015 for the season of OND. Grey color bars indicate the observed Nino3.4 SST anomalies, and blue and orange color bars indicate forecast Nino3.4 SST anomalies in the present climate simulations (pElNino' = pElNino – pClim) and the warmer climate simulations (wElNino' = wElNino – wClim), respectively.

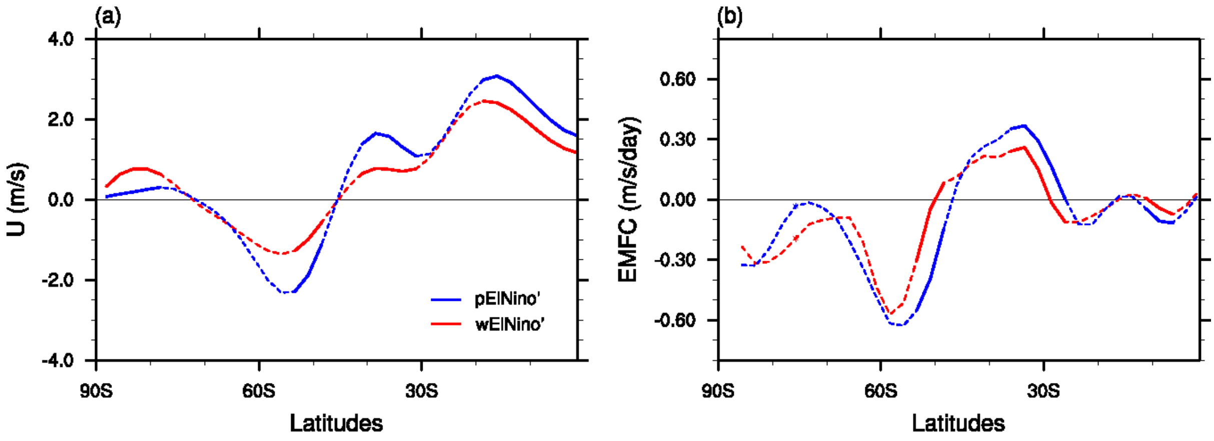

Supplementary Figure S5| Changes of zonal-mean zonal winds and eddy momentum flux convergence associated with pElNino' and wElNino' at the 300 hPa level.

(a) Zonal-mean zonal wind anomalies of pElNino' (blue curve) and wElNino' (red curve); (b) Same as (a) except eddy momentum flux convergence. The thick curves indicate that at a given latitude, the difference between pElNino' and wElNino' is statistically significant at the 5% level, assessed by a two-sided Student t-test with 99 samples of pElNino' and of wElNino'

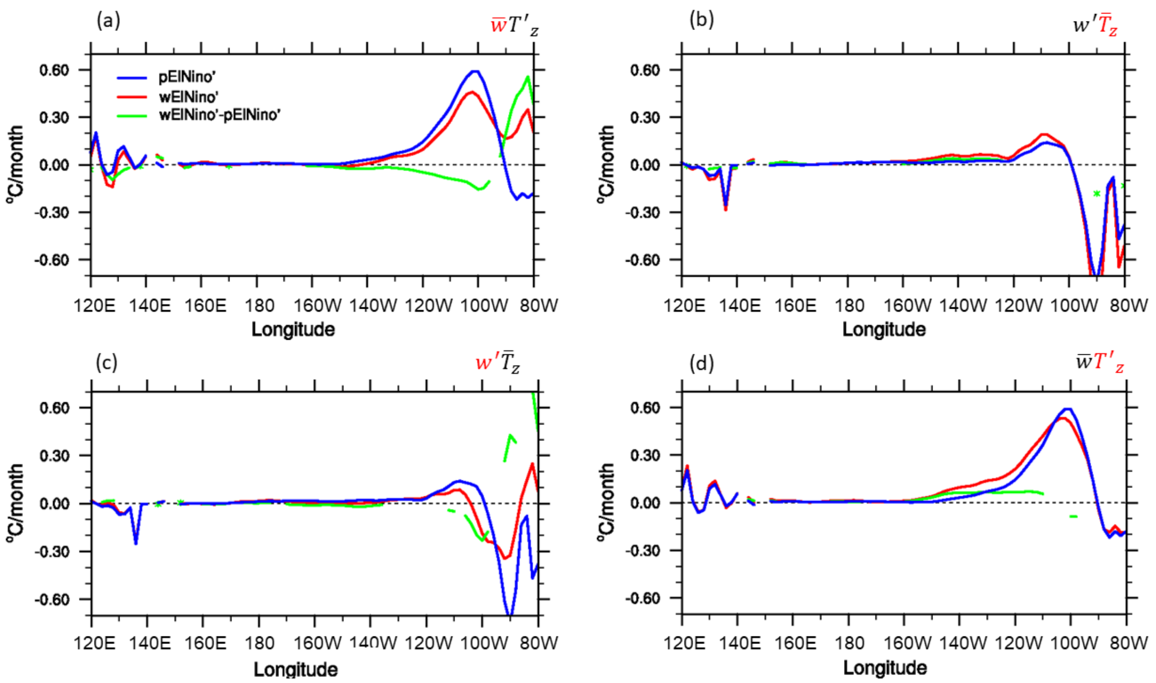

Supplementary Figure S6| Linear thermocline feedback terms decomposed for the contributions of the mean and anomalous upwelling and vertical temperature gradients at the bottom of the ocean mixed layer (45 m) over the tropical Pacific (averaged over 5°S-5°N). Thermocline feedback by (a) the mean ocean upwelling at 45 m ($\overline{w}$) of pClim (blue curve) and wClim (red curve) with the vertical temperature gradients (*T'z*) of pElNino'; (b) the anomalous upwelling of pElNino' (w') with the mean vertical temperature gradients ($\overline{T}_z$) of pClim (blue curve) vs wClim (red curve); (c) the anomalous upwelling (w') of pElNino' (blue curve) vs wElNino' (red curve) with the mean vertical temperature gradient of pClim ($\overline{T}_z$); and (d) the mean upwelling of pClim ($\overline{w}$) with the anomalous vertical temperature gradients (*T'z*) of pElNino' (blue curve) vs wElNino' (red curve). Green curves indicate the differences statistically significant at the 5% level, using two sets of 99 samples.

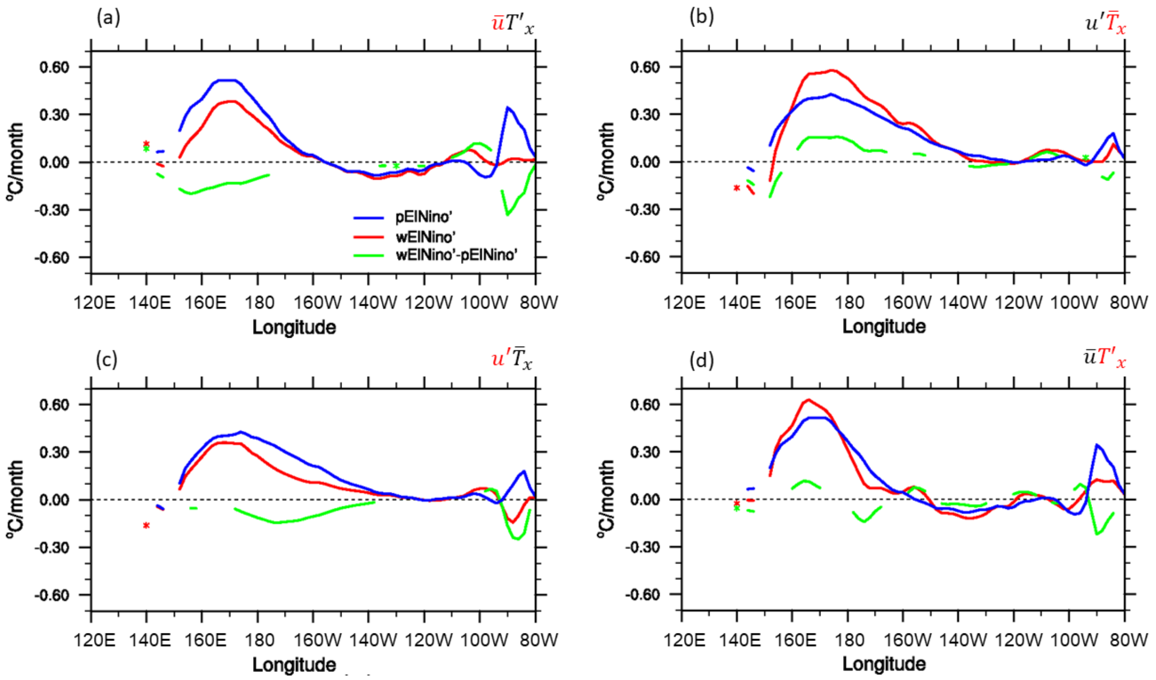

Supplementary Figure S7| Same as Figure S6 except linear zonal advective feedback terms. Linear advective feedback terms are decomposed for the contributions of the mean and anomalous zonal currents ($\bar{u}$, u', respectively) and zonal temperature gradients ($\bar{T}_x$, $T'x$, respectively) averaged in the ocean mixed layer (45 m) over the tropical Pacific (averaged over 5°S-5°N).

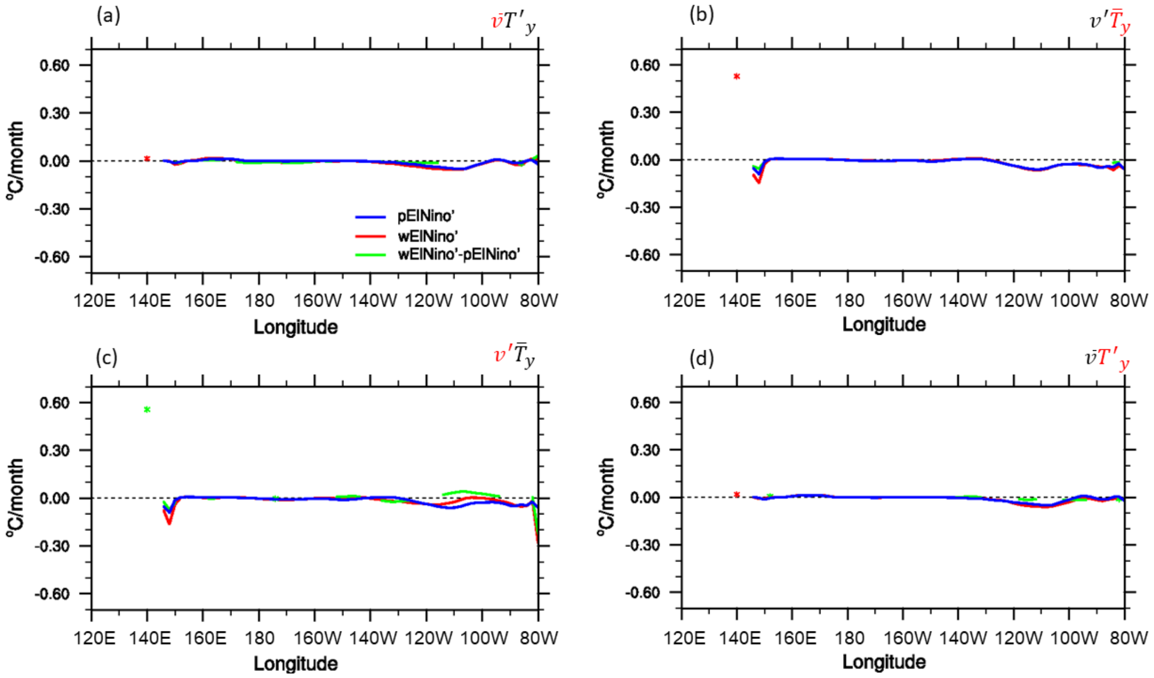

Supplementary Figure S8| Same as Figure S6 except linear meridional advective feedback terms. Linear meridional advective feedback terms are decomposed for the contributions of the mean and anomalous meridional currents ($\bar{v}$, v', respectively) and meridional temperature gradients ($\bar{T}_y$, $T'y$, respectively) averaged in the ocean mixed layer (45 m) over the tropical Pacific (averaged over 5°S-5°N).

Supplementary reference list

1. North, G. R., Bell, T. L., Cahalan, R. F. & Moeng, F. J. Sampling Errors in the Estimation of Empirical Orthogonal Functions. *Mon. Weather Rev.* **110,** 699–706 (1982).

2. Hurrell, J. W., Hack, J. J., Shea, D., Caron, J. M. & Rosinski, J. A new sea surface temperature and sea ice boundary dataset for the community atmosphere model. *J. Clim.* **21,** 5145–5153 (2008).

3. Yin, Y., Alves, O. & Oke, P. R. An Ensemble Ocean Data Assimilation System for Seasonal Prediction. *Mon. Weather Rev.* **139,** 786–808 (2011).
